# The impact of the COVID-19 pandemic on nostalgic social media use

**DOI:** 10.3389/fpsyg.2024.1431184

**Published:** 2024-10-15

**Authors:** Peng Xiang, Lijuan Chen, Fuming Xu, Shasha Du, Mingxuan Liu, Yimeng Zhang, Jiayu Tu, Xiaoyuan Yin

**Affiliations:** ^1^Department of Social Work, Nanjing University of Finance and Economics, Nanjing, China; ^2^The High-Quality Development Evaluation Institute, Nanjing University of Posts and Telecommunications, Nanjing, China; ^3^Faculty of Education, Yunnan Normal University, Kunming, China; ^4^School of Social and Public Administration, Lingnan Normal University, Zhanjiang, China; ^5^College of Philosophy, Jilin University, Changchun, China

**Keywords:** pandemic, nostalgia, social media, nostalgic social media use, Douyin

## Abstract

**Introduction:**

Despite popular speculation that nostalgic social media use skyrocketed during the COVID-19 pandemic, this has yet to be formally investigated in the scientific literature.

**Methods:**

Interrupted time series analysis (ITSA) using a segmented regression model was performed to examine the changes in the weekly volume of searches for nostalgic songs on *Douyin* (the Chinese version of TikTok), as a proxy for nostalgic social media use, before and after the lockdown of Wuhan (signaled the start of the pandemic on a national scale in China).

**Results:**

Across the study period (January 1, 2019–February 28, 2021), an immediate and significant increase in nostalgic social media use was observed when the pandemic initially started (95% CI = [47314.30, 154969.60], *p* < 0.001) compared with the pre-pandemic baseline.

**Discussion:**

This study provides empirical evidence for the impact of the pandemic on nostalgic social media use. It also advances our understanding of the increased usage of social media during the pandemic. Additionally, as nostalgia has drawn increasing attention from media researchers, this study offers methodological insights into the quantification of nostalgia.

## 1 Introduction

Evidence suggests that the usage of social media can facilitate user’s engagement with nostalgia, a sentimental longing for one’s past, contributing to the so-called *nostalgic social media use,* i.e., using social media to engage in nostalgia. On the other hand, since the onset of the coronavirus disease 2019 (COVID-19) pandemic, nostalgia has been shown to function as a psychological resource that helps people get through this unprecedented public crisis. Taken together, it appears intuitively reasonable that the break of the pandemic had led more people to turn to social media to engage in nostalgia. To the best of our knowledge, however, such conjecture has not been well examined in the literature. The current study aimed to address this research gap by determining whether users of *Douyin* (the Chinese version of TikTok), consumed more nostalgic songs after the onset of the pandemic.

### 1.1 Nostalgic social media use: using social media to engage with nostalgia

Social media generally refers to “digital platforms, services, and apps built around the convergence of content sharing, public communication, and interpersonal connection” (Burgess et al., 2017). The usage of social media has been dramatically rising over the years. According to the Digital 2023 Global Overview Report, the number of social media users around the world has grown by about 177% since 2013, reaching 4.76 billion in 2023 (DataReportal, 2023). This means social media has been used by more than 60% of the global population. More importantly, the amount of time for social media use has been constantly increasing. It is estimated that the average time using social media across the various platforms has climbed from 90 min in 2012 to 151 min in 2023 (Statista, 2024). Time spent on social media is not just for connecting with others, since social media has evolved from a platform for socializing to a hub for serving almost all aspects of daily life (e.g., retrieving information, consuming music, online shopping). This means that apart from social motivations, social media use can also be driven by personal motivations. For instance, as music has become more accessible on social media, social media has been the most used channel for people, in particular Gen Z and Millennials to consume music, followed by streaming services dedicated to music (YPulse, 2022).

With the meteoric rise of social media use, people’s ways of behaving have shifted considerably, in particular how we create, experience, and express emotions (Ellis and Tucker, 2020). Nostalgia is such a case. Nostalgia is a bittersweet but predominantly positive emotion resulting from “a sentimental longing or wistful affection for the past” (Pearsall, 1998). This emotion is self-relevant—nostalgic experience typically involves personally meaningful life events, people or settings, but also highly social—the self in nostalgic reveries is often embedded in structures of social relations (Juhl and Biskas, 2022). As such, nostalgia is believed to confer key aspects of psychological wellbeing (e.g., self-discontinuity, meaning in life, social connectedness) (Routledge et al., 2013; Sedikides and Wildschut, 2016), hereby serving as a coping resource in times of crisis. Evidence is accumulating that nostalgia could be the antidote to psychological distresses, such as loneliness (Abeyta et al., 2020; Zhou et al., 2008), boredom (Van Tilburg et al., 2013), pessimism (Kersten et al., 2016), meaninglessness (Routledge et al., 2011), death anxiety (Routledge et al., 2008).

Nostalgia engagement has been facilitated by the usage of social media in many ways. Considering that social media has been becoming a major channel for recording daily life, it is common for users to express and share their nostalgic feelings in the form of text, photographs, or videos by posting on social media (Davalos et al., 2015). Social media platform also tempts users to revisit their “good old days” by looking back at previously posted content (Jacobsen and Beer, 2021). This is true especially when technological features of social media platforms indeed accelerate reminiscing over the past (e.g., Facebook Memories and On This Day on Instagram) (Jungselius and Weilenmann, 2023). Besides, as social media platforms increasingly function as search engines, it can be expected that social media users actively retrieve nostalgia-themed content to consume (e.g., searching for songs of the 1980s on YouTube). Taken together, the coupling of social media use with nostalgia engagement has brought about a special type of social media use, namely nostalgic social media use.

### 1.2 Nostalgia engagement as coping response to the COVID-19 pandemic

The COVID-19 pandemic break has directly or indirectly exposed people to unprecedented psychological threats. In the early days of the pandemic, people across the world were extremely panicked about the coronavirus (Nicomedes and Avila, 2020; Nie et al., 2021). This panic was subsequently compounded with waves of psychological distress (e.g., loneliness, boredom, angst) brought by the prolonged social isolation, resulting from massive-scale lockdowns, stay-at-home orders, or social distancing policies enacted by governments (Glowacz and Schmits, 2020; Luijten et al., 2021; Rossi et al., 2020). Beyond social isolation, there are many drastic lifestyle changes due to the pandemic (e.g., financial hardship, racial discrimination, bereavement) which are detrimental to mental health, and such effects even persist longer than the virus itself (Rossi et al., 2020; Vindegaard and Benros, 2020). In sum, the onset of the pandemic has opened a Pandora’s Box of psychological adversity, and in this case, an individual’s coping strategy is crucial.

As the pandemic and resulting psychological crisis unfold, nostalgia has been proposed as one of the coping resources that are available during this extremely difficult time (Wildschut and Sedikides, 2023). Studies administered in the early days of the pandemic have found that the pandemic was associated with elevated nostalgia. For instance, respondents with greater perceived COVID-19 severity (Xia et al., 2021) or fear of lockdown-related isolation (Wulf et al., 2022) reported higher levels of nostalgic feelings. Loneliness, a common negative experience caused by the pandemic lockdown was shown to be positively related to nostalgia (Huang et al., 2024; Zhou et al., 2022). Furthermore, studies have documented that nostalgia can benefit people through boosting subjective wellbeing (Cho et al., 2024) and counteracting loneliness (Zhou et al., 2022). Besides, nostalgia-based intervention was found to significantly decrease the fear of COVID-19 (Dennis and Ogden, 2022), and foster optimism and vitality (Puente-Díaz and Cavazos-Arroyo, 2021).

### 1.3 Did the COVID-19 pandemic increase nostalgic social media use?

Based on the above-reviewed literature, it is tempting to speculate that the break of the COVID-19 pandemic may have motivated more people to turn to social media to engage in nostalgia. However, to the best of our knowledge, such speculation has not been well examined so far. This research gap is striking because there has been evidence regarding the beneficial role of nostalgic social media use during the pandemic. In a qualitative study on the diverse manifestations of nostalgia-related posts on Facebook, Todorova and Padareva-Ilieva (2021) claimed that posting nostalgia-related content is capable of uniting people, and raising spirit during the COVID-19 crisis. The results of surveys involving U.S. and South Korean samples showed that nostalgic social media use could promote emotional wellbeing via accelerating perceived self-continuity (Lee-Won et al., 2023).

Moreover, some scholars have investigated the impact of the pandemic on nostalgia engagement on non-social media platforms. In Barauskaitė et al. study, for example, the weekly sum of Google searches for nostalgic products was found to be significantly related to weekly COVID-19 cases in the United States (Barauskaitė et al., 2022). Drawing on the data collected from Spotify, a popular music streaming platform, Yeung (2020, 2023) demonstrated that nostalgic songs are consumed more by Spotify’s users from European countries during the lockdown compared with the pre-lockdown period. Although promising, the aforementioned findings are limited in methodological respects. The study of Barauskaitė et al. (2022) is limited by the lack of pre-pandemic benchmarks. Methodological concerns in Yeung’s studies relate to the somewhat arbitrary operationalization of nostalgia engagement. In particular, nostalgia engagement was operationalized by Yeung (2020, 2023) as listening to songs older than 3 or 5 years. Nevertheless, “song age does not equate with a song’s nostalgic resonance” (Huang et al., 2024). Another factor that may undermine Yeung’s findings is that relatively few new songs could be accessed during the COVID-19 pandemic because the music industry has been hit hard since the start of the pandemic (Huang et al., 2024).

## 2 Overview of the present research

The present study aimed to determine whether or not the COVID-19 pandemic has proliferated nostalgic media use in China. As is well known, China is one of the most impacted countries by the pandemic across the globe, and wherein psychological crisis caused by such pandemic is unprecedented in its scale and magnitude. Based on this, together with accumulating evidence showing the palliative role of nostalgia, it can be assumed that Chinese social media users might be especially motivated to turn to nostalgia to seek psychological solace. However, the majority of studies in this area were conducted in a Western cultural context, and to our knowledge, few studies have been done in China to link nostalgia engagement and the pandemic (for exception, Zhou et al., 2022), let alone to directly examine the impact of the pandemic on nostalgic social media use.

Building on the prior works (Huang et al., 2024; Yeung, 2023), nostalgic social media use in this study was operationalized as the consumption of nostalgic songs on Donyin, one of the most popular social media platforms in China. To address methodological concerns in Yeung’s studies (Yeung, 2020, 2023), inspired by relevant research (Xiang and Xu, 2023), search volume of keywords related to nostalgic songs on Douyin was recruited to measure nostalgic song consumption. Douyin, known as the Chinese version of TikTok, is a video-sharing social media platform developed by a Chinese company called ByteDance in 2016. It was initially established as a music-centered platform and allowed users to post video clips lasting from 15 s to 1 min on this platform. And now, it has evolved into a bigger platform that supports 15-min-long videos with various genres. Since its emergence, Douyin has quickly climbed to one of the most popular social media platforms in China, with over 700 million daily active users as of 2023 (Apps, 2024). People on Douyin achieve social interaction with others through liking, commenting, sharing, or following. They are also encouraged to build their social network by participating in viral campaigns on Douyin, such as Trending challenges—create and share videos that follow a specific theme or trend. Moreover, the social features of Douyin are further reinforced by its algorithm-driven content recommendation system. Due to this system, users are continually exposed to content that is tailored to their interests and preferences, which in turn leads to the formation of user communities with like-minded individuals. In such communities, more frequent interactions among users are possible.

More importantly, Douyin was selected over other social platforms because it has publicly provided a weighted sum of the search volume for keywords since 2020 (hereafter, Douyin Search Index). Douyin Search Index is constructed based on the absolute retrieval volume of keywords produced by the user’s information retrieval behavior on Douyin. Although the exact algorithm is not accessible to the public because of trade secrets, it is claimed that Douyin Search Index can capture directly user’s interest directly, with a higher index indicating a higher level of interest in the relevant topic.

## 3 Methods

The weekly volume of searches for keywords regarding nostalgic songs powered by Douyin Search Index, was utilized to quantify nostalgic social media use. Based on a previous study (Xiang and Xu, 2023), five keywords related to nostalgic songs were selected, i.e., 怀旧金曲 (golden songs), 怀旧老歌 (old songs), 怀旧歌曲 (nostalgic songs), 经典歌曲 (classics songs), 经典老歌 (retro songs). These keywords are the same semantically, referring to songs that can evoke nostalgic feelings, and have been used indiscriminately by Chinese. The Cronbach’s *α* coefficient was 0.92 for these keywords, reflecting a strong internal consistency among them. Therefore, the sum of these five keywords was constructed as a measurement of nostalgic social media use in this study, with higher values indicating a higher level of nostalgia engagement on social media. The data we collected ranges from January 1, 2019, through February 28, 2021 (i.e., 113 weeks). According to The State Council Information Office of China, following the lockdown of Wuhan, the epicenter of the COVID-19 outbreak in China, 31 provincial-level regions launched the highest-level public health emergency response to the pandemic within 1 week (The State Council Information Office of China, 2020). And under the first-level public health emergency response, partial- or full-lockdown measures were carried out across China. Thus, the lockdown of Wuhan was associated with nationwide influence and has been regarded as a marker of the beginning of COVID-19 in China in previous studies (e.g., Wu et al., 2023; Yang et al., 2021). In the current study, the weeks up to and including the week Wuhan’s lockdown occurred were defined as the pre-COVID-19 period (January 1, 2019–January 27, 2020), and the weeks onwards as the post-COVID-19 period (January 28, 2020–February 28, 2021).

Unlike previous studies (e.g., Barauskaitė et al., 2022), the study timeframe in this study did not exclusively focus on a certain period of COVID-19 lockdown. The present study is based on the premise that nostalgic social media use occurred as a coping response to psychological distress caused by the pandemic. Notably, the negative psychological impacts of the pandemic can continue even after the lift of lockdown measures. With this in mind, the present study attempted to determine the possible effect of the pandemic on nostalgic social media use beyond the certain lockdown period. Consensus was achieved among authors regarding this study period.

Interrupted time series analysis (ITSA) using a segmented regression model was performed to examine the changes in the weekly volume of searches for nostalgic songs on Douyin before and after the start of the COVID-19 pandemic. The ITSA is a quasi-experimental method for isolating the effect of an intervention (e.g., policy change, major public crisis) on a time series of outcome measures, by controlling for secular trends of outcomes before interventions (Penfold and Zhang, 2013). In the segmented regression model, a time series is divided into pre- and post-intervention segments. This method has two parameters of interest: the level and slope of the two segments. A change in level indicates the short-term effect of interventions, and a change in slope suggests the long-term effect of interventions (Bernal et al., 2017). To eliminate the confounding influences of seasonality and other long-term trends in the weekly volume of searches for nostalgic songs on Douyin, Fourier terms (pairs of sine and cosine functions) were included in models (Bhaskaran et al., 2013).

## 4 Results

### 4.1 Descriptive analysis

The raw data revealed a distant pattern of nostalgic social media use between the periods before and after the COVID-19 outbreak. The weekly nostalgic social media use across the pre-pandemic period ranged from 12,805 to 249,629, with an average of 104898.66. In the during-pandemic period, the weekly nostalgic social media use ranged from 195,831 to 544,267, with an average of 333612.89.

### 4.2 Interrupted time-series analysis

**Figure 1:**
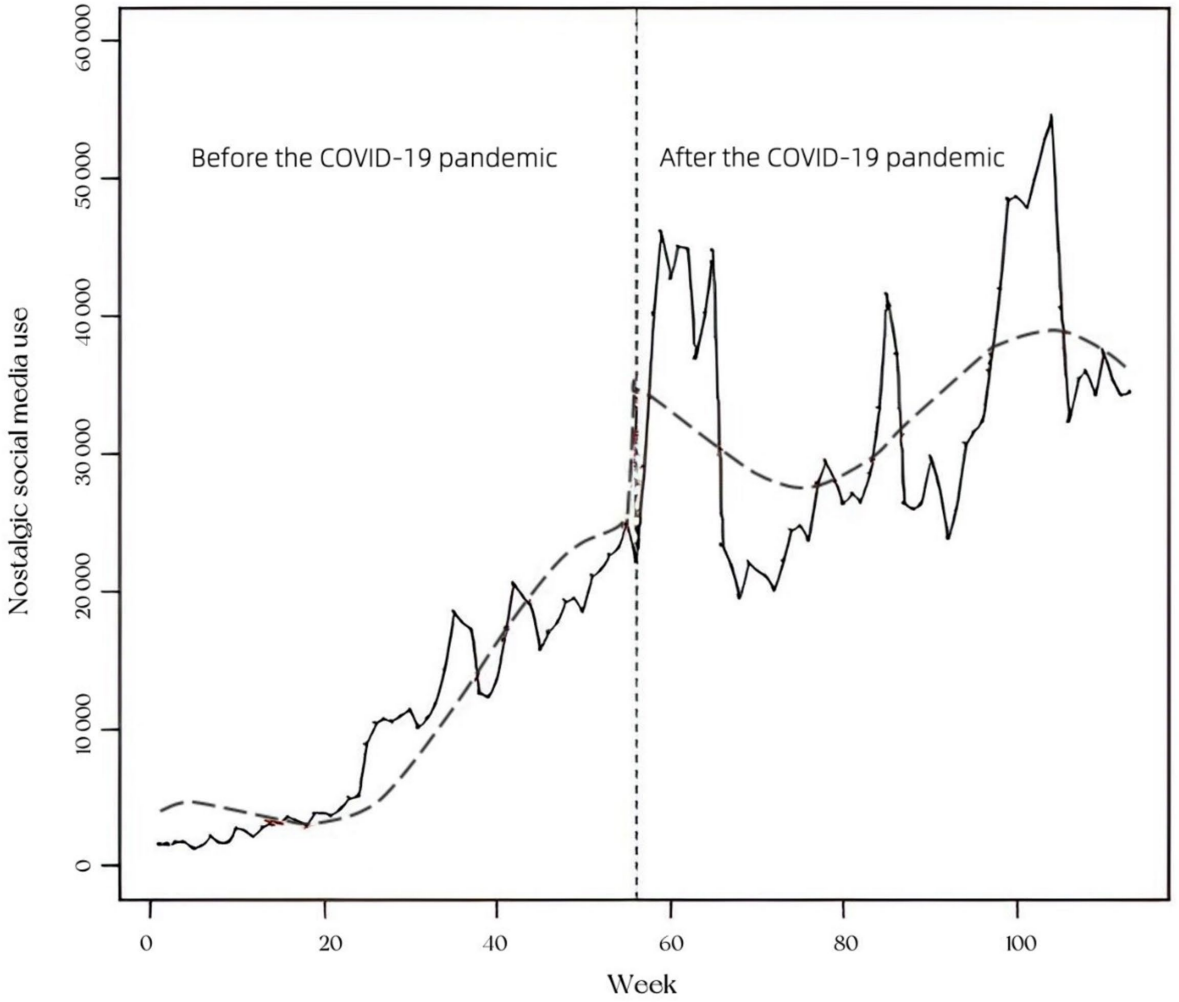
Nostalgic social media use before and after the COVID-19 pandemic. The dotted curve line is predicted trend based on the seasonally adjusted regression model. The vertical dotted line represents the introduction of the pandemic.

As Figure 1 shows, there was a steady increase in nostalgic social media use during the pre-pandemic period. Results of the ITSA suggest that nostalgic social media use climbed 3953.60 per week on average across the pre-pandemic period (95% CI = [2791.90, 5115.40], *p* < 0.01; Results of the ITSA are presented in Table 1). As assumed, an immediate and significant surge of nostalgic social media use was observed when the pandemic started. The level of nostalgic social media use increased by 101142.00 upon the introduction of the pandemic (95% CI = [47314.30, 154969.60], *p* < 0.01). However, the increase peace of nostalgic social media use slowed down after the pandemic break, dropping from 3953.60 per week in the pre-pandemic period to 697.70 per week during the period of the pandemic. These results indicate that the onset of the COVID-19 pandemic was indeed associated with the abrupt increase in nostalgic social media use.

**Table 1:** Interrupted time-series regression estimates for the nostalgic social media use before and after the COVID-19 pandemic.

Parameters	Estimate	SE	*p*-value	95%CI
Slope before the pandemic	3953.60	586.00	< 0.001	2791.90 to 5115.40
Level change after the pandemic	101142.00	27153.00	< 0.001	47314.30 to 154969.60
Slope change after the pandemic	−3255.90	9813.80	< 0.001	−4618.40 to −1893.40

## 5 General discussion

In this study, we extracted the data from Douyin, and empirically investigated the possible impact of the COVID-19 pandemic on nostalgic social media use in China. Across the study period (January 1, 2019–February 28, 2021), we observed a significant increase in nostalgic social media use immediately when the pandemic started. This change could be attributed to the fact that the initial phase of the pandemic was extremely impactful, resulting in the desire for nostalgia being particularly strong. It is worth noting that during the period of the pandemic, nostalgic social media use continued to grow, but at a slower pace compared to the pre-pandemic period. The reason behind it may be that hundreds of millions of people had been pushed into a state of chronic stress due to the seesawed pandemic (Qi et al., 2021), and unsurprisingly nostalgia lingered.

Notably, the impact of the pandemic onset is not linear. As is depicted in Figure 1, nostalgic social media use exhibited wild fluctuations after the start of COVID-19: besides the first peak that has been discussed earlier, the other two peaks also emerged during this period. The second peak occurred in mid-August 2020. After the lifting of the 76-day lockdown of Wuhan, the pandemic in China experienced a fluctuant downward trend from mid-April to the end of July 2020. But it dramatically resurged around mid-August, and hence many cities (e.g., Daqing, Sanya) underwent full or partial lockdown. The third peak took place around the end of December 2020, coinciding with a new wave of explosive growth of the pandemic during that period. It was reported that newly confirmed indigenous cases in China increased dramatically in December 2020. Unsurprisingly, lockdown measures were carried out in dozens of Chinese megacities at that time (e.g., Xian, Dalian, Shenyang). It should be noted that since China implemented severe policies to control the COVID-19 epidemic, the local pandemic and resulting measures can matter nationally (Xu et al., 2020).

In addition to the rationale discussed in the section of the introduction, the results reported here may also be interpreted by confounding effects brought by a sudden rise in social media use itself after the pandemic break. As the pandemic and the resulting measures taken to prevent it have upended nearly every aspect of daily life, people have had to turn to social media to maintain their daily routines. Unsurprisingly, a remarkable rise in social media use was observed after the onset of the pandemic: more people registered as users of social media, and more time spent using social media (Luo et al., 2021; Werling et al., 2021). This fact may simultaneously contribute to a significant rise in nostalgia engagement on social media. Unfortunately, data on the number of Douyin users or time for using Douyin pre- and during the pandemic are not available, which in turn hinders us from determining its confounding effect. Yet this methodological limitation could be justified by previous studies (Ho et al., 2021; Van Kessel et al., 2023), it is still advisable that results of this study should be interpreted with caution.

The current study contributes to the literature in several ways. First of all, this study empirically determines the impact of the COVID-19 pandemic on nostalgic social media use with a convincing method. Although existing findings indicate that social media users may be more likely to engage in nostalgia (e.g., consuming nostalgic cultural products) during the COVID-19 pandemic than usual, direct evidence still lacks. The most relevant studies to our study are conducted by Yeung (2020, 2023), who found that the COVID-19 pandemic lockdown has led to a dramatically increased consumption of nostalgic songs (operationalized as the song released 3 or more years before collection time). However, Yeung’s findings have been criticized due to the relatively arbitrary operationalization of nostalgic songs in his studies (Huang et al., 2024). This issue was addressed in the present study by employing the real-time search volume of nostalgic songs on Douyin as the nostalgia engagement on social media. By doing so, to the best of our knowledge, this study is the first to quantify the significance of the pandemic in increasing nostalgic social media use.

Second, the findings of this study are expected to advance our understanding of the increased usage of social media during the pandemic. Given that the usage of social media has become an integral part of daily lives, it is unsurprisingly that significant change in social media usage has been reported since the start of the COVID-19 pandemic (Bailey et al., 2022; Nilsson et al., 2022; Thygesen et al., 2021). Nevertheless, this megatrend has been largely attributed to instrumental motivations—people relied heavily on social media to stay informed about the disease (Muñiz-Velázquez et al., 2021; Neely et al., 2021), maintain socially connected (Midgley et al., 2022) or seek entertainment during the pandemic (Bowden-Green et al., 2021). The present study here suggests another possibility behind the dramatic increases in social media use during the COVID-19 pandemic, that is, people may turn to social media for self-rehabilitation endeavors like engaging in nostalgia.

Third, this study contributes to the emerging literature on the interlinkage of nostalgia and social media use. Recent years have seen an increased scientific interest in the fact that nostalgia engagement has been plat formed by the usage of social media (Jacobsen and Beer, 2021). Indeed, feelings of nostalgia could be triggered by social media, approached through social media, or expressed on social media. More importantly, nostalgia-related behaviors of social media users produce large data sets of content, which in turn can provide a new avenue to explore nostalgia engagement on a large scale (Davalos et al., 2015). Notably, it is argued that digital data can address methodological flaws related to self-reported data of outcomes of interest (e.g., recall bias, social desirability effect). Although promising, many challenges exist in using data obtained from social media platforms to study nostalgia, and one of these challenges involves how to quantify nostalgia. In this regard, the approach to define and measure nostalgia engagement employed in the present study provides an alternative for future research on this topic.

As a retrospective investigation, the present study has some practical implications for future public crisis management. The empirical findings presented here underscore engaging in nostalgia as a self-coping mechanism during times of crisis like the COVID-19 pandemic. This informs policy-makers that implementing nostalgia-based intervention could be on their crisis management agenda. More importantly, the present study suggests that such endeavor is expected to be made via social media platforms.

Several limitations should be acknowledged in the present study. First, data on outcomes of interest were only extracted from Douyin platform, which means that people who do not have access to Douyin have been excluded from this study. This may undermine the credibility of the finding presented here. Nevertheless, the massive popularity of Douyin in China has to some extent made up for this problem. Second, nostalgic social media use is a multifaceted concept that manifests in various forms, but it was only operationalized as nostalgic song consumption on social media in this study. This represents a possible limitation and points to a future direction. Future studies could examine whether the present findings could be generalized to other operationalizations of nostalgic social media use. Third, although we observed there is a marked increase in nostalgic social media use immediately after the outbreak of the pandemic, it is impossible to identify what sort of people have contributed to this result due to aggregated-level analysis. This limitation may further hinder us from ascertaining which groups should be targeted with tailored information in nostalgia-based intervention.

## 6 Conclusion

By operationalizing nostalgic social media use as a search volume of keywords related to nostalgic songs on Douyin, we examined changes in nostalgic social media use between the pre-and during-pandemic period. We found that the onset of the COVID-19 pandemic was associated with an immediate increase in nostalgic social media use. The result of this study provides quantified evidence to verify the significance of the pandemic in increasing nostalgic social media use. It highlighted that during the pandemic, social media served as a valuable platform for people to seek psychological solace. Additionally, as nostalgia has drawn increasing attention from media researchers, this study offers methodological insights into the quantification of nostalgia. Further research with more sophisticated is required to confirm our results.

## Data Availability

The raw data supporting the conclusions of this article will be made available by the authors, without undue reservation.
